# Comparison of Subjective Image Quality of Mobile C-Arms Equipped with a-Si, CMOS or IGZO Flat-Panel Detectors for Intraoperative Fluoroscopy

**DOI:** 10.3390/jimaging12080337

**Published:** 2026-07-24

**Authors:** Fenna Brunken, Benno Bullert, Robert Brauweiler, Paul A. Grützner, Sven Y. Vetter, Nils Beisemann

**Affiliations:** 1 BG Klinik Ludwigshafen, Department for Orthopaedics and Trauma Surgery at Heidelberg University, Ludwig-Guttmann-Str. 13, 67071 Ludwigshafen, Germany; 2 Siemens Healthineers AG, Siemensstr. 3, 91301 Forchheim, Germany

**Keywords:** C-arm, flat-panel detector, a-Si, CMOS, IGZO

## Abstract

Different flat-panel detector technologies are available for intraoperative fluoroscopy. This study compared orthopedic and trauma surgeons’ preference and subjective image quality among mobile C-arm systems equipped with amorphous silicon (a-Si), complementary metal oxide semiconductor (CMOS) or indium gallium zinc oxide (IGZO) detectors. Fluoroscopic imaging was performed on four human specimens at four anatomic locations at pulse rates of 1/s and 10/s in low- and high-dose settings using three C-arm systems. Subjective image quality was rated by two observers on 5-point Likert scales. Pairwise forced-choice comparisons of images with identical acquisition parameters were analyzed using a Bradley–Terry model. Across all images, CMOS- and IGZO-based systems were preferred over the a-Si-based system in 91.8% and 90.4% of comparisons, respectively (ORs 9.53 and 11.20; both *p* < 0.001). No significant overall preference was observed between the IGZO- and CMOS-equipped systems. Subjective image quality ratings were significantly higher for CMOS- and IGZO-based systems compared with the a-Si-based system, particularly for overall image quality and perceived noise, while no consistent differences in image quality were found between CMOS- and IGZO-based systems. Overall, the CMOS- and IGZO-based systems evaluated in this study were preferred over the a-Si-based system and achieved superior subjective image quality ratings.

## 1. Introduction

Intraoperative fluoroscopy is widely used in orthopedic and trauma surgery to assess fracture reduction and implant positioning. Flat-panel detectors (FPDs) offer several advantages, including improved spatial resolution and the potential for reduced radiation exposure compared to traditional image intensifiers and have become the standard for fluoroscopy in orthopedic and trauma surgery [1,2,3].

Different types of FPDs are used in mobile C-arms. The most common detectors are based on amorphous silicon (a-Si), complementary metal oxide semiconductor (CMOS) or indium gallium zinc oxide (IGZO) sensors.

All three detector types can be implemented as indirect-conversion systems using structured cesium iodide (CsI) scintillators but differ in their readout architectures. Conventional a-Si and IGZO FPDs are typically based on active matrix thin-film transistor arrays, while CMOS FPDs commonly use active pixel sensors [4,5]. The charge mobility of a-Si is lower compared to CMOS and IGZO, which may contribute to higher electronic noise and slower readout rates [5,6,7]. Additionally, CMOS technology enables smaller pixel pitches, potentially improving spatial resolution [6,8].

Currently, a-Si detectors are widely used for intraoperative fluoroscopy. However, technical evaluations have demonstrated that CMOS detectors exhibit lower electronic noise and reduced image lag in comparison to a-Si detectors [5]. Additionally, CMOS FPDs demonstrate higher detective quantum efficiency (DQE) under low-dose conditions and at high spatial frequencies, while DQE is slightly reduced at moderate to high-dose levels compared with a-Si detectors [5]. Previous studies have also demonstrated a reduction in electronic noise and an increase in mid-frequency DQE for IGZO compared to a-Si detectors [9]. However, most available evidence regarding the image quality of different detectors for fluoroscopy is derived from technical analysis, and previous studies have generally been limited to pairwise comparisons of detector technologies [5,9].

There is limited data on the clinical assessment of image quality for the different FPDs available for mobile C-arms. Consistent with previous studies on the technical performance, Abiola et al. reported that 2D fluoroscopy images acquired with CMOS detectors were preferred by interventional radiologists over images acquired with a-Si detectors at low-dose settings [10]. However, IGZO detectors were not included in that study and direct comparisons of the perceived image quality of all three detector types used for intraoperative fluoroscopy are lacking.

In this study, the subjective image quality of three commercially available mobile C-arms equipped with either an a-Si, CMOS or IGZO detector was compared for fluoroscopy in four different anatomic regions in human specimens.

## 2. Materials and Methods

### 2.1. Image Acquisition

In this study, three mobile C-arm systems equipped with either an a-Si, CMOS or IGZO FPD were compared. The a-Si detector was used in the Cios Fusion system. The CMOS and IGZO detectors were used in Cios Flow/Flow.neo systems (all Siemens Healthineers, Erlangen, Germany). The evaluated C-arm systems are based on the same mechanical platform with an identical source-to-image distance. All detectors had a field of view of 30 × 30 cm. The a-Si FPD had a native pixel size of 0.194 mm, the CMOS FPD of 0.152 mm and the IGZO FPD of 0.109 mm. All C-arms were operated in 2 × 2 binning mode, resulting in an effective pixel size of 0.388 mm (a-Si), 0.304 mm (CMOS) and 0.218 mm (IGZO). The physical detector matrix sizes were 1536 × 1536 pixels for the a-Si FPD, 1952 × 1952 pixels for the CMOS FPD and 2776 × 2776 pixels for the IGZO FPD. In 2 × 2 binning mode, the corresponding matrix sizes were 768 × 768 (a-Si), 976 × 976 (CMOS) and 1388 × 1388 (IGZO) pixels, respectively. All systems used a bit depth of 16 bits. The total filtration was 3 mm Al equivalent at 75 kVp + 0.1 mm Cu for all three systems. No additional filters were used in this study. Before data collection, all three C-arm systems underwent quality control according to the manufacturer’s standards, consistent with the procedures applied during routine service intervals.

Fluoroscopic images were acquired at the upper ankle, distal radius, knee joint and lumbar spine of four fresh-frozen whole-body human specimens. The mean BMI of specimens was 23.04 kg/m^2^ (range: 20.66–29.26 kg/m^2^) (see Table 1). The study was approved by the responsible ethics committee (application number 2024-17893). The specimens were thawed at room temperature for 24 h prior to use. There was no radiographic evidence of pre-existing fractures or implants in the regions assessed.

Images were taken in anteroposterior and lateral projections at all locations. To ensure reproducibility across all C-arm systems, 3D-printed positioning guides were used. The correct positioning was confirmed by two orthopedic and trauma surgery residents.

Fluoroscopic images were acquired at pulse rates of 1/s and 10/s in high-dose and low-dose settings with each C-arm. The reported dose represents the incident air kerma per pulse at the detector entrance. Dose levels were set to 0.015 µGy/p for low-dose and 0.026 µGy/p for high-dose using manufacturer-defined presets and were kept constant across the three systems. The corresponding pulse dose values were obtained from the settings of the respective C-arm. Before the study, all systems were dose-calibrated at the detector surface using an external dosimeter in accordance with the manufacturer’s instructions.

Automatic exposure control was enabled for all acquisitions, adjusting tube voltage and tube current-time product according to the selected dose levels. To allow comparability, a single fluoroscopic frame obtained one second after acquisition start was used for further analysis, independent of the applied pulse rate. Before acquisition of the evaluated frames, fluoroscopy was performed at the same position to allow the automatic exposure control to stabilize after any change in the imaging scene. This approach was intended to ensure that the evaluated acquisitions primarily reflected the effects of pulse rate and temporal averaging. The one-second interval was selected to limit the influence of progressive image stabilization associated with temporal noise filtering to allow an assessment of image quality at low pulse rates without prolonging fluoroscopy time. Default fluoroscopic image processing was applied on all systems. Temporal noise filtering was enabled using identical settings (k-factor = 8) across all C-arm systems. Collimation was kept constant for each anatomical region across all C-arm systems.

### 2.2. Image Assessment

**Figure 1:**
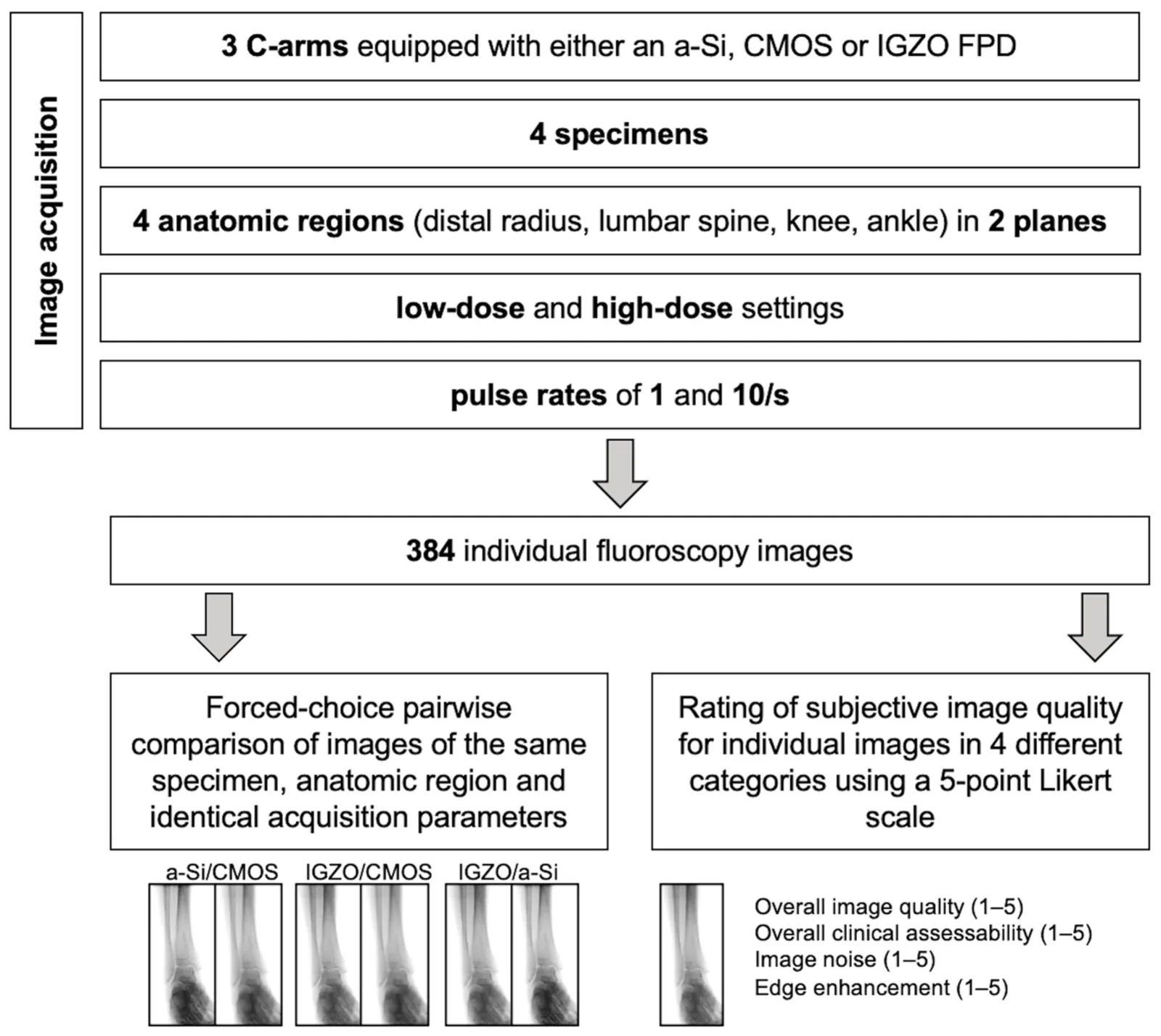
Study design.

The images were presented to two orthopedic and trauma surgery residents in their third and fourth years of residency, respectively. Both observers had previous experience with the subjective assessment of 2D and 3D fluoroscopic image quality using Likert scales and were provided with a table defining the rating criteria for each category before the evaluation. Observers were blinded to the C-arm system and pulse rate/dose setting used. Images were presented using a standard DICOM viewer (Horos 4.0.1, Horos Project, Annapolis, MD, USA) and observers were allowed to use the image manipulation tools available (e.g., contrast, zoom). All datasets were anonymized and randomly sorted. Forced-choice pairwise comparisons of images with identical acquisition parameters of the same region and specimen were conducted. Images were displayed side by side on the same monitor with a screen resolution of 1920 × 1080 pixels and observers were asked to choose which image they would prefer in the intraoperative setting. After a two-week washout period to reduce potential recall bias [11,12], the same observers were asked to rate the overall image quality, clinical assessability, perceived image noise and edge enhancement for each individual image on a 5-point Likert scale (see Figure 1 and Table 2).

### 2.3. Statistics

Data were analyzed using Prism 10 (GraphPad, San Diego, CA, USA) and R 4.5.0 (R Core Team, Vienna, Austria).

Normal distribution was tested by Shapiro–Wilk Test. Descriptive statistics are presented as medians with interquartile range (IQR) for non-normally distributed data. Multiple comparisons of non-normally distributed data were analyzed using a Friedman test with Dunn’s post hoc correction. To evaluate the surgeons’ preference among images acquired with the different C-arm systems, forced-choice pairwise comparisons were analyzed using a Bradley–Terry model. Stratified models were additionally computed for anatomical regions (spine vs. extremities) and for imaging conditions (pulse rate and dose level) to assess potential interaction effects between C-arm system preference and image acquisition settings. Interrater reliability was assessed using weighted Cohen’s Kappa for each image quality category (overall quality, clinical assessability, noise, edge enhancement). *p*-values < 0.05 were considered statistically significant.

## 3. Results

**Figure 2:**
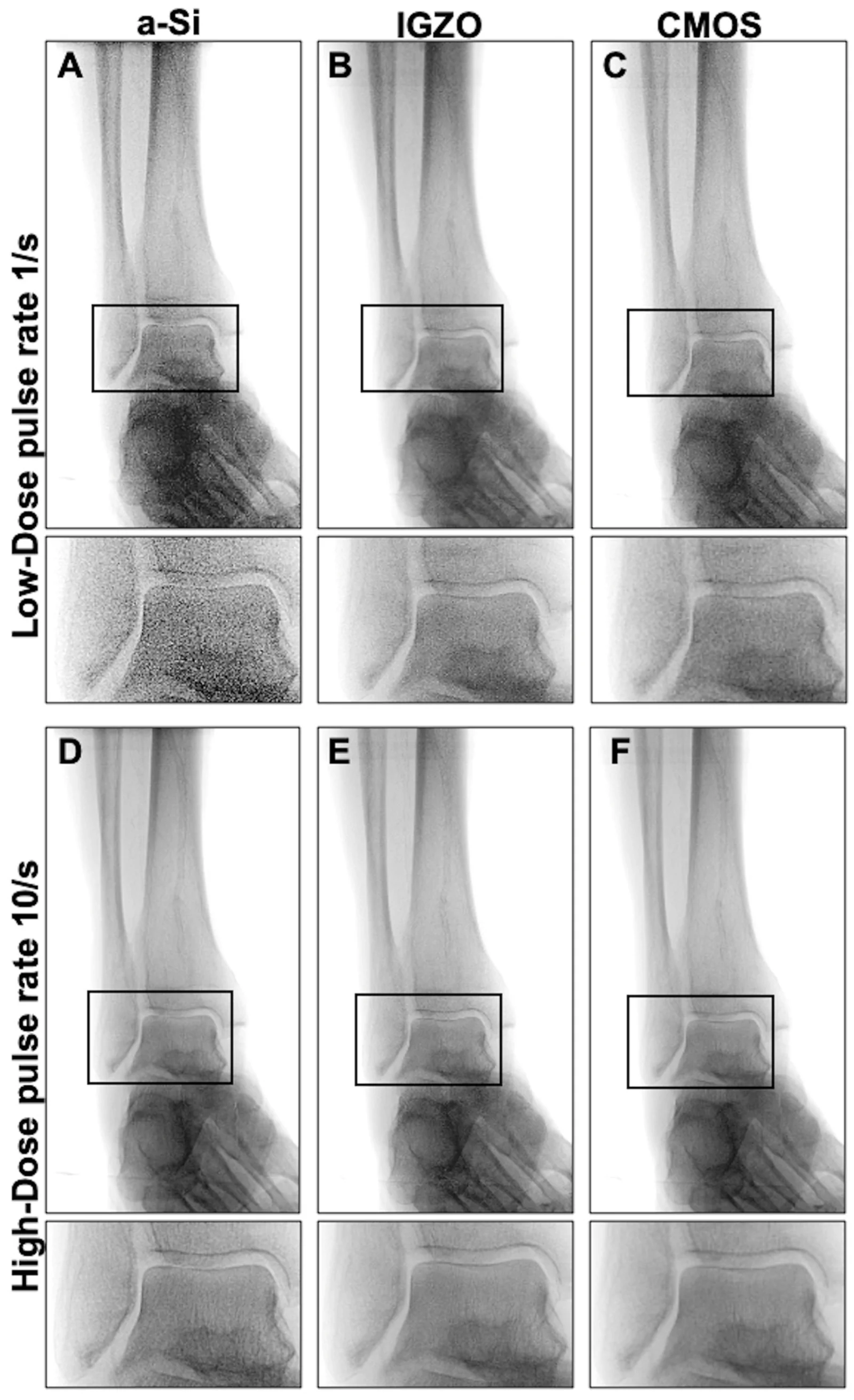
Examples of fluoroscopy images of the upper ankle taken with C-arms equipped with a-Si (**A**,**D**), IGZO (**B**,**E**) and CMOS (**C**,**F**) detectors with a pulse rate of 1/s and low-dose settings (**A**–**C**) or with a pulse rate of 10/s and high-dose settings (**D**–**F**).

**Figure 3:**
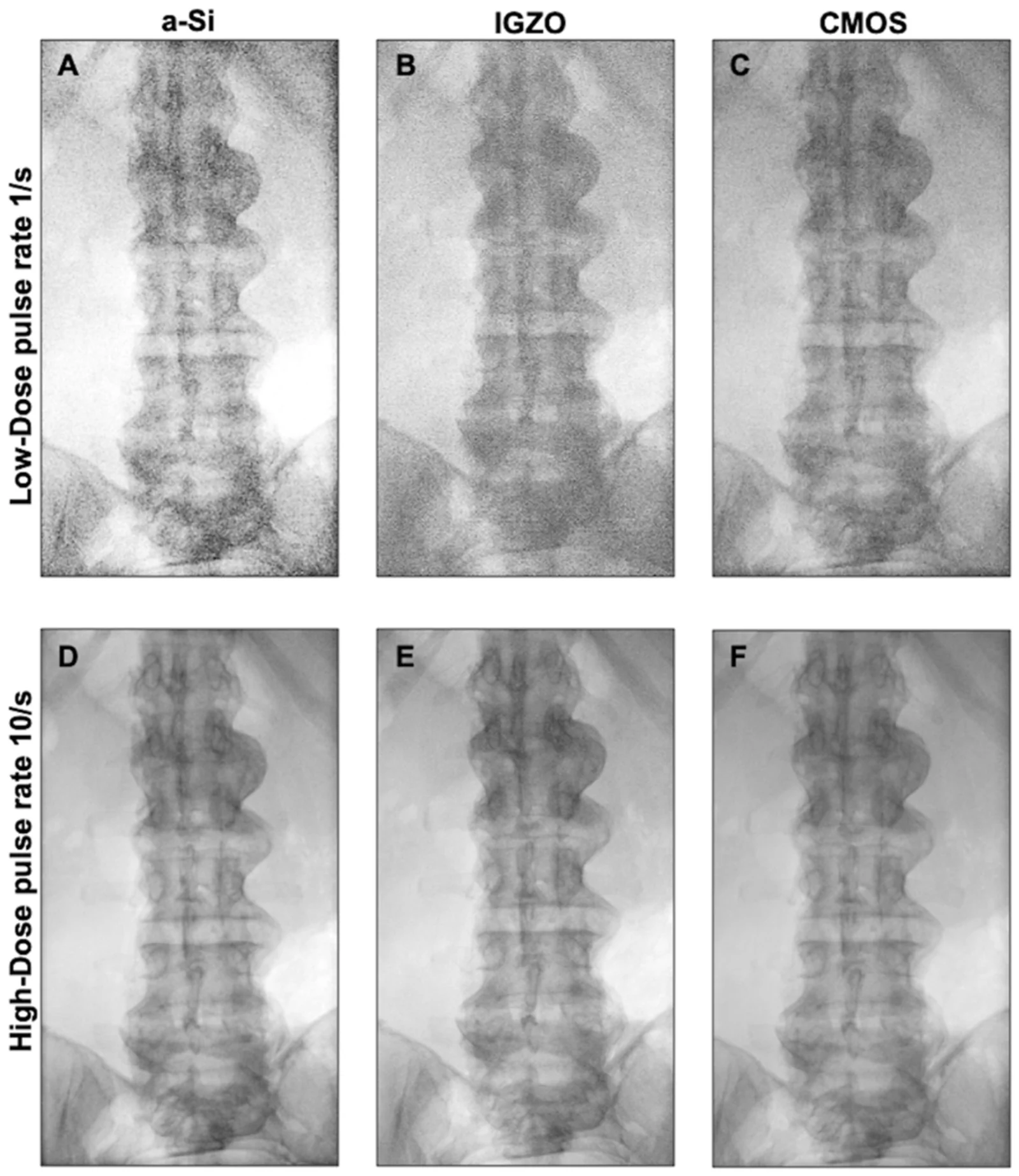
Examples of fluoroscopy images of the lumbar spine for the three C-arms with a-Si (**A**,**D**), IGZO (**B**,**E**) and CMOS (**C**,**F**) detectors. The upper row displays images acquired with a pulse rate of 1/s and low-dose settings; the lower row shows images acquired with a pulse rate of 10/s in high-dose settings.

A total of 384 individual images were evaluated by two independent observers. Additionally, 384 forced-choice pairwise comparisons were performed for images acquired under identical conditions with the three C-arm systems. Representative examples of the images analyzed are shown in Figure 2 and Figure 3.

### 3.1. Preference in Pairwise Comparisons

**Figure 4:**
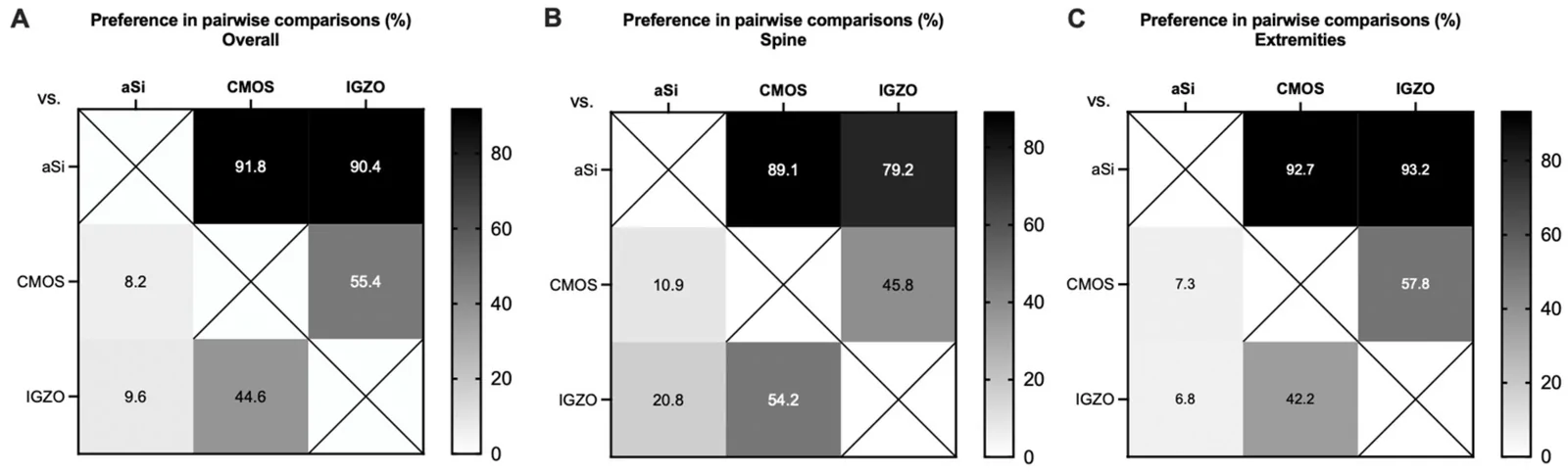
Heatmaps of pairwise forced-choice comparisons among the three C-arm systems equipped with different detectors (a-Si, IGZO, CMOS). Values represent the percentage of comparisons in which the column system (detector type shown in the column label) was preferred over the row system (detector type shown in the row label). (**A**): Overall. (**B**): Spine. (**C**): Extremities.

Overall, images acquired with the C-arm equipped with a CMOS detector were preferred over those obtained with the a-Si-based system in 91.8% of cases, and the IGZO-based system was preferred over the a-Si-based system in 90.4% of comparisons. Images acquired with the IGZO-based system were favored over the CMOS-based system in 55.4% of pairwise comparisons (Figure 4).

Analysis using a Bradley–Terry model confirmed that systems equipped with CMOS and IGZO FPDs were significantly preferred over the a-Si-based system (both *p* < 0.001), while the difference between IGZO-based and CMOS-based systems was not statistically significant (*p* = 0.18) (see Table 3).

For spinal images, the system equipped with the CMOS FPD was preferred over the system with an IGZO FPD in 54.2% of cases; however, this difference was not statistically significant (*p* = 0.233). Both CMOS-based (89.1%) and IGZO-based systems (79.2%) were significantly favored over the a-Si-based system (both *p* < 0.001) (see Figure 4 and Table 4).

In extremity images, the IGZO-based system was preferred over the CMOS-based system in 57.8% of pairwise comparisons. Images acquired with the C-arms equipped with CMOS and IGZO FPDs were selected over those obtained with the a-Si-based system in 92.7% and 93.2% of cases, respectively (see Figure 4). The Bradley–Terry model confirmed that both CMOS- and IGZO-equipped systems were significantly preferred over the a-Si-based system (both *p* < 0.001). For extremity images, the difference between the systems equipped with IGZO and CMOS FPDs was statistically significant (*p* = 0.036) (see Table 5). Across all images, observers agreed on the preferred image in 77.5% of pairwise comparisons.

**Figure 5:**
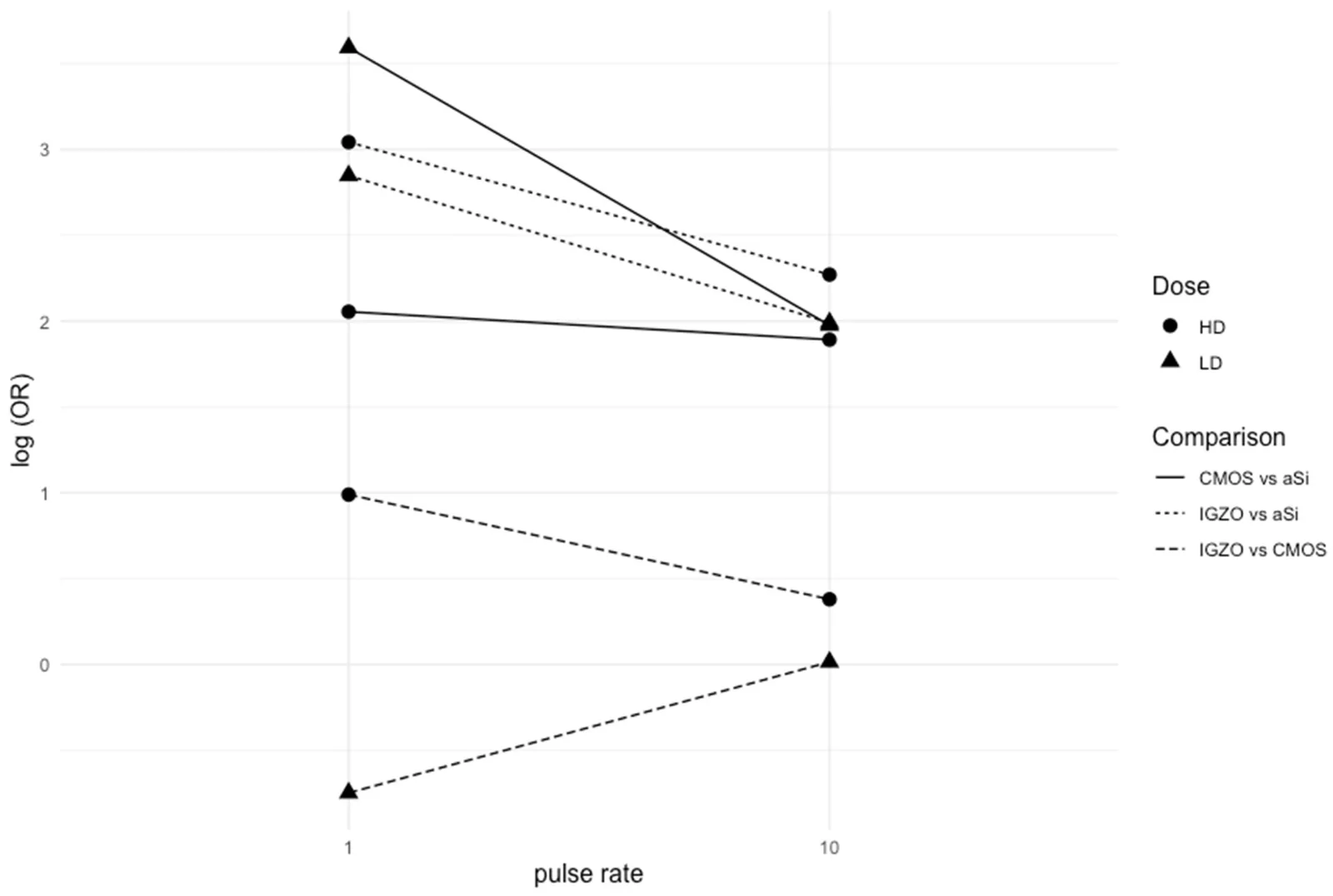
Preference in pairwise comparisons is shown as estimated log odds ratios from the Bradley–Terry model for the different C-arm systems for pulse rates of 1/s and 10/s under high-dose (HD) and low-dose (LD) conditions.

The influence of pulse rate and dose setting on preference in pairwise comparisons is shown in Figure 5. In low-dose settings, the preference of the CMOS-based system over the a-Si-based system was significantly reduced at higher pulse rates (10/s) compared with low pulse rates (1/s) (*p* = 0.03), with a similar but non-significant trend observed for the preference of the IGZO-based system over the a-Si-based system. For the comparison of IGZO- and CMOS-based systems in low-dose settings, a relative preference for the CMOS-based system was observed at a pulse rate of 1/s, while there was no preference for either system with a pulse rate of 10/s. The IGZO-based system was preferred over the CMOS-based system in high-dose settings, especially at a pulse rate of 1/s (Table A1).

### 3.2. Subjective Image Quality

Overall image quality was rated significantly higher for the C-arm systems equipped with CMOS and IGZO detectors compared to the a-Si-based system, while no significant difference was observed between the CMOS- and IGZO-equipped systems. Subjective image noise was rated significantly worse for the a-Si-based system compared with the CMOS- and IGZO-based systems (*p* < 0.0001). Detailed results for subjective image quality ratings are shown in Table 6.

For the subgroup analysis of spinal images, subjective image quality ratings showed significant differences between the systems only for overall image quality, clinical assessability and edge enhancement at low-dose settings with a pulse rate of 1/s and for image noise at high-dose settings with a pulse rate of 1/s (Table A3).

For extremity images, subjective image quality ratings were significantly higher for the CMOS- and IGZO-based systems compared to the a-Si-based system across most pulse rate and dose settings (see Table A2). No significant differences in subjective image quality were observed between the C-arms equipped with CMOS and IGZO FPDs across image quality categories in post hoc testing.

Inter-rater reliability assessed using weighted Cohen’s Kappa showed moderate agreement for the ratings of edge enhancement (κ = 0.52) and overall image quality (κ = 0.60), and substantial agreement for clinical assessability (κ = 0.63) and image noise (κ = 0.66). Exact agreement for Likert scale ratings between the observers was 38.6% for overall image quality, 39.4% for clinical assessability, 47.3% for image noise, and 34.6% for edge enhancement.

## 4. Discussion

Currently, a-Si detectors are widely used for digital radiography, conventional intraoperative fluoroscopy and cone beam CT (CBCT) [6,13,14,15,16,17]. However, a-Si detectors have demonstrated reduced performance parameters, especially under low-dose conditions [5,9]. Previous studies have reported technical advantages of CMOS and IGZO detectors over a-Si detectors in terms of reduced electronic noise and increased DQE [5,7,9,18]. However, a direct comparison of CMOS and IGZO detectors is lacking. The present study demonstrated a higher observer preference and improved subjective image quality ratings for C-arm systems equipped with CMOS and IGZO detectors compared with the a-Si-based system. Final image quality is influenced by several factors and cannot be attributed to detector technology alone. Although comparable acquisition presets and identical temporal-filter settings were used, not all image acquisition and post-processing parameters could be standardized. Automatic exposure control determines parameters such as tube voltage and tube current-time product according to object attenuation and the selected acquisition preset. Although automatic exposure control was enabled for all acquisitions and similar dose presets were used, the underlying control algorithms were proprietary and could not be standardized. Similarly, the post-processing algorithms were system-specific and may have affected perceived noise, sharpness, contrast and overall image appearance. Therefore, the findings should be interpreted as a comparison of complete C-arm systems rather than an isolated comparison of detector technologies.

### 4.1. CMOS/a-Si Comparison

Sheth et al. performed a technical analysis and subjective image quality comparison of CMOS and a-Si detectors in mobile C-arms. Electronic noise was reduced in CMOS compared to a-Si detectors. Additionally, DQE was higher for CMOS compared to a-Si at low-dose levels while a-Si showed a higher DQE at high-dose levels and low frequencies [5].

Despite the technical advantages, no perceived difference was observed for tasks in orthopedic surgery, although the visibility of fine details in low-dose fluoroscopy was rated superior for CMOS compared to a-Si [5].

Abiola et al. compared the preference of interventional radiologists for specific tasks between systems equipped with CMOS and a-Si detectors for 2D and 3D imaging. Fluoroscopy images acquired using a CMOS detector were preferred over a-Si, especially in low-dose settings, while no significant difference was observed with high-dose settings [10]. The present study also demonstrated a stronger preference for CMOS- over a-Si-based systems in low-dose compared to high-dose settings, especially at low pulse rates. At higher pulse rates, this difference was reduced, possibly due to the influence of temporal filtering, which may decrease perceived image noise [19]. Since only frames acquired one second after the initiation of fluoroscopy were analyzed, the number of frames available for temporal filtering differed among the pulse rate settings. At a pulse rate of 1/s, temporal noise reduction may not have reached a steady state, possibly contributing to higher perceived image noise at low pulse rates [19,20,21]. Accordingly, subjective ratings of image noise for individual images in this study were also lower at higher pulse rates. Overall, these results suggest that systems equipped with CMOS detectors might be preferred over a-Si-based systems in low-dose settings, consistent with previously reported technical findings [5].

### 4.2. IGZO/a-Si Comparison

Regarding the comparison of IGZO and a-Si detectors, a 1.7-fold reduction in electronic noise and an increase in mid-frequency DQE have been reported for IGZO FPDs at similar effective pixel sizes [9]. Previous studies concluded that IGZO detectors may offer advantages over a-Si, particularly under low-dose conditions or at higher frame rates [9]. In the present study, the system equipped with an IGZO detector was consistently preferred over the a-Si-based system in pairwise comparisons and demonstrated superior subjective ratings, independent of dose setting and pulse rate.

### 4.3. IGZO/CMOS Comparison

Direct comparisons of IGZO and CMOS detectors are currently lacking. Previous studies indicate that the electronic noise performance of IGZO detectors is superior to that of a-Si detectors but inferior to that of CMOS detectors [9]. However, at higher exposure levels, CMOS detectors have demonstrated reduced DQE compared to a-Si, while IGZO detectors showed equivalent or increased DQE under these conditions [5,9]. A-Si detectors exhibit a lower charge mobility compared to CMOS and IGZO FPDs, contributing to higher electronic noise and slow readout rates [5,6,7].

No significant overall preference was observed between the IGZO- and CMOS-equipped systems in the present study. For extremity images, the IGZO-based system was preferred over the CMOS-based system, however the effect was modest (OR 1.33, 95% CI 1.02–1.74).

According to manufacturer specifications, the IGZO-based system evaluated in the present study provides a higher spatial resolution in overview fluoroscopy mode (2.2 lp/mm) compared with the CMOS-based system (1.8 lp/mm), which may partly relate to differences in effective pixel size. In addition, manufacturer-reported DQE values are higher for the IGZO detector (approximately 80%) than for the CMOS detector (approximately 72%). These technical characteristics may have contributed to the observed preference for IGZO over CMOS in extremity imaging, where the visibility of small details may be particularly important. However, this explanation remains speculative because the systems also differed in image processing and the independent contributions of spatial resolution, DQE and other system-specific factors could not be determined.

### 4.4. Clinical Implications

In intraoperative fluoroscopy, minimizing radiation exposure is important, as both patients and operating room staff are exposed. Previous studies have shown an increased prevalence of malignancies among orthopedic surgeons [22,23,24]. Therefore, optimizing image quality under low-dose conditions represents an important objective in the development of C-arm systems. Radiation exposure to patients and medical staff can be reduced by using pulsed fluoroscopy and lower pulse rates [25,26]. Pulse rates between 5 and 15/s have been reported for orthopedic procedures [27,28].

Median clinical-assessability ratings indicated that images were sufficient for intraoperative assessment under all evaluated conditions except for the a-Si- and IGZO-equipped systems at a pulse rate of 1/s under low-dose conditions. This may suggest that differences between the systems become more clinically apparent under low-dose conditions at low pulse rates. The reduced subjective image quality observed for the C-arm equipped with an a-Si detector in low-dose settings in this study is consistent with the higher susceptibility of a-Si detectors to electronic noise at low exposure levels reported in prior technical evaluations [5,9]. However, the present study did not evaluate whether these differences affected diagnostic accuracy or intraoperative decision-making.

It should be noted that additional factors, such as system cost, long-term detector durability, and CBCT performance, may also influence the choice of C-arm system. CMOS detectors have been reported to exhibit lower radiation resistance compared with a-Si detectors [6].

This study only evaluated 2D imaging. Previous studies have demonstrated advantages of CMOS FPDs over a-Si FPDs for CBCT in terms of reduced noise and improved detectability of small features [5,15,29]. IGZO FPDs have also demonstrated improved 3D imaging performance compared to a-Si FPDs [9].

### 4.5. Limitations

This study was conducted using human specimens to allow for repeated imaging without radiation exposure to patients. However, this experimental setup does not fully reflect the clinical use of fluoroscopy in orthopedic and trauma surgery and further studies evaluating the effect on surgical decision making based on assessment of fracture reduction and implant positions are needed.

Image quality and observer preference were compared between three commercially available C-arm systems equipped with different detectors. Although the systems share a similar design and comparable presets were used, observed differences in image quality may reflect not only detector characteristics but also additional system-specific factors, including automatic exposure control and image post-processing. Therefore, the findings of this study represent a comparison of C-arm systems rather than an isolated assessment of detector performance. Additionally, all evaluated C-arms were produced by a single manufacturer, which limits the generalizability of the findings to systems from other manufacturers because of potential differences in system design, automatic exposure control and image-processing algorithms.

Pixel size differed across the three systems and may have contributed to differences in perceived image quality. The effective pixel size of the IGZO detector was smaller (0.218 mm) compared to the effective pixel sizes of the CMOS (0.304 mm) and a-Si (0.388 mm) detectors. These variations may have contributed to differences in spatial resolution and perceived image noise between the C-arm systems. However, their independent contribution could not be determined.

This study evaluated only subjective image quality. No objective image-quality metrics, such as modulation transfer function, DQE or noise power spectrum, were obtained as independent references for the observed differences between imaging conditions, limiting direct comparison with previous technical assessments.

Image assessment was performed by two trauma surgery residents, experienced in interpreting intraoperative fluoroscopic images and subjective image assessment. Although the observers represented the intended clinical users, the limited number and experience range of observers restrict the generalizability of the findings.

Observers agreed on the preferred image in pairwise comparisons in 77.5% of cases.

Inter-rater reliability for the Likert-scale ratings ranged from moderate to substantial, likely reflecting individual preferences in subjective image-quality assessment. Still, this variability should be considered when interpreting the results, particularly given the limited number of observers.

No formal sample-size or power calculation was performed because of the exploratory nature of the study and the absence of sufficient preliminary data. The findings should therefore be considered exploratory and require confirmation in larger studies involving additional specimens and observers with different levels of experience.

Bradley–Terry models have been applied in radiological studies to analyze forced-choice pairwise comparisons of image quality [30,31,32,33]. Previous studies have suggested that forced-choice comparisons are superior in detecting small differences in perceived image quality and may provide better inter- and intraobserver agreement compared to Likert scales [12,34,35]. Still, Likert scales are frequently used to assess subjective image quality of fluoroscopic images [36,37]. Therefore, pairwise forced-choice comparisons were used to assess preference in this study, while Likert scale ratings were used to evaluate specific image quality categories.

## 5. Conclusions

In this study, the C-arm systems equipped with CMOS and IGZO detectors were preferred over the a-Si-based system and were rated higher for subjective image quality. Overall, no statistically significant difference was observed between the CMOS- and IGZO-based systems. However, subjective image quality reflects overall system performance and is not determined by detector technology alone.

## Figures and Tables

**Table 1:** Specimen characteristics.

Specimen	Sex	Age (Years)	BMI (kg/m^2^)	Height (cm)	Weight (kg)	Primary Cause of Death
1	M	93	20.67	175	64	Acute Hypoxic Respiratory Failure
2	F	79	21.58	157	54	Cardiac arrest
3	M	83	20.66	178	65	Senile Degeneration of the Brain
4	F	84	29.26	157	73	Unspecified natural causes

**Table 2:** Criteria for the subjective rating of image quality on a 5-point Likert scale.

Score	Overall Image Quality	Overall Clinical Assessability	Image Noise	Edge Enhancement
1	Reduced	Unacceptable, assessment not possible	Unacceptable	Reduced
2	Slightly reduced	Limited	Above average	Slightly reduced
3	Acceptable	Moderate, sufficient for intraoperative assessment	Average	Acceptable
4	Good	Good	Minimal	Good
5	Excellent	Excellent	Not perceptible	Excellent

**Table 3:** Bradley–Terry Model Results for overall pairwise comparisons.

Comparison	Log (OR) ± SE	Odds Ratio (95% CI)	*p*
**CMOS vs. a-Si**	2.25 ± 0.17	9.53 (6.87–13.21)	**<0.001**
**IGZO vs. a-Si**	2.42 ± 0.17	11.20 (8.01–15.66)	**<0.001**
**IGZO vs. CMOS**	0.16 ± 0.12	1.18 (0.93–1.49)	0.178

**Table 4:** Bradley–Terry Model Results for pairwise comparisons of spinal images.

Comparison	Log (OR) ± SE	Odds Ratio (95% CI)	*p*
**CMOS vs. a-Si**	1.86 ± 0.29	6.41 (3.63–11.35)	**<0.001**
**IGZO vs. a-Si**	1.56 ± 0.30	4.73 (2.65–8.44)	**<0.001**
**IGZO vs. CMOS**	−0.31 ± 0.26	0.74 (0.45–1.22)	0.233

**Table 5:** Bradley–Terry Model Results for pairwise comparisons of extremity images.

Comparison	Log (OR) ± SE	Odds Ratio (95% CI)	*p*
**CMOS vs. a-Si**	2.45 ± 0.21	11.55 (7.68–17.39)	**<0.001**
**IGZO vs. a-Si**	2.74 ± 0.21	15.40 (10.13–23.43)	**<0.001**
**IGZO vs. CMOS**	0.29 ± 0.14	1.33 (1.02–1.74)	**0.036**

**Table 6:** Ratings of different criteria for subjective image quality are shown as median (IQR). PR: Pulse rate, LD: low-dose, HD: high-dose.

	PR	Dose	a-Si	CMOS	IGZO	*p*-Value
**Overall image quality**	**1**	**LD**	1 (1–2)	2 (2–2)	2 (2–2)	<0.0001
	**10**	**LD**	3 (3–4)	4 (3–4)	4 (3–4)	0.0006
	**1**	**HD**	2 (2–3)	3 (2–3)	3 (3–3)	<0.0001
	**10**	**HD**	4 (4–4)	4 (4–5)	4 (4–5)	<0.0001
**Clinical assessability**	**1**	**LD**	2 (2–2)	3 (2–3)	2 (2–3)	<0.0001
	**10**	**LD**	4 (3.75–4)	4 (4–4)	4 (4–4)	0.27
	**1**	**HD**	3 (2–3)	3 (3–3.25)	3 (3–3)	0.0001
	**10**	**HD**	4 (4–5)	5 (4–5)	5 (4–5)	0.0006
**Image noise**	**1**	**LD**	1 (1–1)	2 (2–2)	2 (2–2)	<0.0001
	**10**	**LD**	3 (3–3)	4 (3–4)	4 (4–4)	<0.0001
	**1**	**HD**	2 (2–2)	3 (2–3)	3 (2–3)	<0.0001
	**10**	**HD**	4 (3.75–4)	4 (4–5)	5 (4–5)	<0.0001
**Edge enhancement**	**1**	**LD**	2 (1–2)	2.5 (2–3)	2 (2–3)	<0.0001
	**10**	**LD**	4 (3–4)	4 (4–4)	4 (4–4)	0.04
	**1**	**HD**	3 (2–3)	3 (3–3)	3 (3–3)	0.02
	**10**	**HD**	4 (4–5)	4 (4–5)	4 (4–5)	0.26

## Data Availability

The data presented in this study are available from the corresponding author upon reasonable request. The data are not publicly available due to ethical and institutional restrictions regarding data obtained from human specimens.

## Abbreviations

The following abbreviations are used in this manuscript:

a-Si	amorphous silicon
BMI	Body Mass Index
CMOS	complementary metal oxide semiconductor
DQE	detective quantum efficiency
FPD	Flat-Panel Detector
IGZO	indium gallium zinc oxide
HD	High-dose
LD	Low-dose
PR	Pulse rate

## Appendix A

**Table A1:** Bradley–Terry model results for pairwise comparisons stratified by pulse rate and dose setting.

	Comparison	Log (OR)	SE	*p*-Value	OR (95% CI)
PR1 LD	CMOS vs. a-Si	3.59	0.49	<0.0001	36.40 (13.95–94.96)
	IGZO vs. a-Si	2.85	0.47	<0.0001	17.25 (6.91–43.07)
	IGZO vs. CMOS	−0.75	0.27	0.0049	0.47 (0.28–0.80)
PR1 HD	CMOS vs. a-Si	2.06	0.34	<0.0001	7.80 (4.00–15.24)
	IGZO vs. a-Si	3.04	0.38	<0.0001	20.98 (9.88–44.52)
	IGZO vs. CMOS	0.99	0.27	0.0003	2.69 (1.58–4.56)
PR10 LD	CMOS vs. a-Si	1.98	0.30	<0.0001	7.22 (4.03–12.93)
	IGZO vs. a-Si	1.99	0.30	<0.0001	7.32 (4.06–13.20)
	IGZO vs. CMOS	0.01	0.23	0.9520	1.01 (0.64–1.60)
PR10 HD	CMOS vs. a-Si	1.89	0.30	<0.0001	6.63 (3.68–11.96)
	IGZO vs. a-Si	2.27	0.32	<0.0001	9.69 (5.20–18.04)
	IGZO vs. CMOS	0.38	0.24	0.1130	1.46 (0.91–2.33)

**Table A2:** Ratings of different criteria for subjective image quality of extremity images shown as median (IQR). PR: Pulse rate, LD: low-dose, HD: high-dose.

	PR	Dose	a-Si	CMOS	IGZO	*p*-Value
**Overall image quality**	**1**	**LD**	1 (1–2)	2 (2–2)	2 (2–2)	<0.0001
	**10**	**LD**	3 (3–4)	4 (3–4)	4 (3–4)	0.012
	**1**	**HD**	2 (2–3)	3 (2.25–3)	3 (3–3)	<0.0001
	**10**	**HD**	4 (4–5)	4.5 (4–5)	4.5 (4–5)	0.0001
**Clinical assessability**	**1**	**LD**	2 (2–2)	3 (2–3)	2.5 (2–3)	0.0004
	**10**	**LD**	4 (4–4)	4 (4–4.75)	4 (4–4)	0.61
	**1**	**HD**	3 (2.25–3)	3 (3–4)	3 (3–3.75)	0.0006
	**10**	**HD**	4 (4–5)	5 (4.25–5)	5 (4–5)	0.0014
**Image noise**	**1**	**LD**	1 (1–1)	2 (2–2)	2 (2–2)	<0.0001
	**10**	**LD**	3 (3–3)	4 (3–4)	4 (4–4)	<0.0001
	**1**	**HD**	2 (2–2)	3 (2–3)	3 (2–3)	<0.0001
	**10**	**HD**	4 (3.25–4)	5 (4–5)	5 (4–5)	<0.0001
**Edge enhancement**	**1**	**LD**	2 (2–2)	3 (2–3)	3 (2–3)	0.002
	**10**	**LD**	4 (4–4)	4 (4–4)	4 (4–4)	0.06
	**1**	**HD**	3 (2.25–3)	3 (3–3)	3 (3–3)	0.03
	**10**	**HD**	4 (4–5)	4.5 (4–5)	4 (4–5)	0.26

**Table A3:** Ratings of different criteria for subjective image quality of spinal images shown as median (IQR). PR: Pulse rate, LD: low-dose, HD: high-dose.

	PR	Dose	a-Si	CMOS	IGZO	*p*-Value
**Overall image quality**	**1**	**LD**	1 (1–1)	2 (1.75–2.25)	2 (1–2)	0.012
	**10**	**LD**	3 (3–3)	4 (3–4)	3.5 (3–4)	0.07
	**1**	**HD**	2 (2–2.25)	3 (2–3)	3 (2–3)	0.11
	**10**	**HD**	4 (4–4)	4 (4–4.25)	4 (4–4.25)	>0.99
**Clinical assessability**	**1**	**LD**	1 (1–2)	2.5 (2–3)	2 (2–3)	0.004
	**10**	**LD**	3.5 (3–4)	4 (3.75–4)	4 (3–4)	0.67
	**1**	**HD**	2.5 (2–3)	3 (2.75–3)	3 (2.75–3)	0.33
	**10**	**HD**	4 (4–5)	4.5 (4–5)	4.5 (4–5)	>0.99
**Image noise**	**1**	**LD**	1 (1–1)	1.5 (1–2)	1.5 (1–2)	0.11
	**10**	**LD**	3 (3–4)	3.5 (3–4)	4 (3–4)	0.67
	**1**	**HD**	2 (1–2)	2 (2–2.25)	2.5 (2–3)	0.02
	**10**	**HD**	4 (3.75–5)	4 (4–4.25)	4 (4–5)	>0.99
**Edge enhancement**	**1**	**LD**	1 (1–2)	2 (1.75–3)	2 (1.75–2.25)	0.02
	**10**	**LD**	3.5 (3–4.25)	4 (4–4)	4 (3.75–4)	0.81
	**1**	**HD**	2.5 (2–3)	2.5 (2–3)	2.5 (2–3.35)	>0.99
	**10**	**HD**	4 (4–5)	4 (4–5)	4 (4–5)	>0.99
